# Development of Adversity Quotient (AQ) index of pre-service teachers in Institute of Teacher Education (IPG)

**DOI:** 10.3389/fpubh.2022.940323

**Published:** 2022-09-29

**Authors:** Rafidah Mohd Adnan, Mohd Effendi Ewan Mohd Matore

**Affiliations:** ^1^Sekolah Kebangsaan (SK) Bangi, Kajang, Selangor, Malaysia; ^2^Research Centre of Education Leadership and Policy, Faculty of Education, National University of Malaysia, Bangi, Malaysia

**Keywords:** Adversity Quotient, index, pre-service, teachers, Institute of Teacher Education

## Abstract

The study aims to develop the Adversity Quotient (AQ) index of pre-service teachers during practicum training. The study also has assessed psychometric characteristics using the Rasch model. The original contribution is by addressing gaps of measuring AQ accurately among pre-service teachers through index at the Institute of Teacher Education (IPG) that not been widely explored. The research design entails a survey with a quantitative approach through questionnaires. The four main constructs of AQ comprises Control, Ownership, Reach, and Endurance (CORE model). This study involves several key procedures such as challenge identification, expert validity, item development, and psychometric testing of items before developing the index. A total of 96 items were produced and piloted over 159 pre-service teachers. Findings from the pilot study showed 54 items that met all assumptions from the Rasch model such as item fit, unidimensionality, local independence, reliability, and separation index. The actual study was conducted on 542 pre-service teachers from five Malaysian Institutes of Teacher Education (IPG) in the Central Zone through stratified random sampling. The data were analyzed using SPSS version 26.0 and WINSTEPS version 3.71.0.1. The findings showed the 46.86% of practicum pre-service teachers have a moderately high AQ index with 74.80. The Control and Ownership recorded a high level AQ index with 77.30 and 77.10, respectively, while Reach and Endurance were at a moderate level AQ index with 73.20 and 71.50. The AQ index of male pre-service teachers is higher (76.29) than the female (74.09). It can be seen that eighth semester pre-service teachers is higher (75.28) than the sixth semester (74.38). The Science (SN) field recorded as a highest index (80.45), while the Visual Arts Education (PSV) field has a lowest index score (70.29). Further studies can be done by reviewing the pre-service teacher development program by empowering the reach and endurance aspects to ensure that the future teachers are resilient to challenges.

## Introduction

Over the past few decades, the field of education in Malaysia has declined the perception that teachers not only deliver knowledge but they also manage to become holistically quality teachers (1). Professional teachers will continue to work tirelessly to improve their own performance, including the performance of society and the country. One of the groups of teachers' worth paying attention to is the future teachers or also known as pre-service teachers. Quality pre-service teachers are important in educating better-quality students. However, the effort in producing quality teachers is often accompanied by various issues and problems. Among the prevalent factors that prevent pre-service teachers from excelling in academia and practical training includes the various forms of problems in training by pre-service teachers vary from seven areas such as administrative support, cooperating teachers, student supervisors, peers, students, related tasks, and learning environment (2). Due to the challenges in schools that they have never experienced before, pre-service teachers tend to face self-adjustment problems (3). These pre-service teachers are often busy and exhausted as a result of all activities outside the practicum sessions that the teachers are obliged to follow. Thus, the time constraint with additional workload has made practicum training a complicated and unsettling one (4).

Mental disorders and stress are also a challenge for pre-service teachers. As opined by (2), pre-service teachers find greater difficulties in the top three areas namely students, related tasks, and peers. They suffer from confusion so much that the practicum practice at school feels extensively tormenting. This is due to their weakness in dealing with challenges, especially the failure to adapt to the burden of many tasks. Pre-service teachers who fail to prepare themselves with the skills and abilities to face changes in the world of education tend to feel stressed and worried (5, 6). Pre-service teachers with a low level of challenge control will always have feelings of dissatisfaction and rebelliousness, weak spirit, and inadequate effort; they are also easy to give up, panic quickly, and do not have high creative and innovation power. All of these are due to stress, nervousness, and too much obedience to the school management (7). Such emotions will, in turn, lead to changes in the pre-service teachers' personality types and these changes may affect their commitment to the teaching profession. In addition, some pre-service teachers are also less motivated, not as committed to the assigned tasks, and do not inquire as much information due to their own attitude (8).

However, in facing these challenges, the ability of pre-service teachers in the aspects of self-control, self-management, and social skills is low such that they are less prominent in their attitude as leaders but prefer to be followers. Based on (9), there are three problems that are often faced by student-teachers related to students' bad attitudes and their learning motivation, including disrespecting the teacher, sleep during the class and lack of participation. The teachers' resilience level is also low, in addition to their lack of social skills. Self-resilience plays an important role in enabling teachers to respond positively, for instance, to challenging employment situations. Teacher resilience, defined as the ability to withstand natural sources of stress and discouragements in teaching as a difficult profession, is critical in all education since it can create numerous positive outcomes (10).

In this regard, the teachers need intelligence called Adversity Quotient or AQ, which refers to a person's ability and spirit to continue facing life's challenges efficiently with critical ability and also a robust predictor of a person's success (11). Adversity Quotient (AQ) is defined by Stoltz (12) as an individual's ability to struggle with a challenge, difficulty, or problem at hand, as well as turning it into a golden opportunity to succeed. In the context of the current study, the term “AQ” is referred to as a measurement of pre-service teachers' ability to meet challenges, overcome existing challenges, and subsequently turn these into an opportunity to achieve success. Success in this context refers to achievement in practicum training.

Therefore, the four AQ constructs measured in this study are control, ownership, reach, and endurance. This construct had proven empirically applied by several researchers recently (13, 14). Recent studies on AQ are heavily influenced by positive psychology (8) that focusing on the identification of resources that protect individuals from experiencing adverse effects due to life challenges. Studies have also shown that the AQ levels of individuals tend to change over their lifetime (15). Besides, past research in the context of education has found that most teachers experience difficulties in changing their routines to adapt to changes (2, 4–6, 9, 16–18). From pre-service teachers perspective, they are low-skilled in overcoming life's challenges because lack of vision, mission, and true meaning of life (19). Their ability to deal with such challenges or AQ demonstrates a clear need to associate AQ with these future teachers. Literature that consistent with pre-service teacher context are very limited. Research by (20) show that AQ gives positive influence to the development of mathematical understanding ability of pre-service mathematics teacher with the influence of 57.3 percent. While, Adversity Quotient (AQ) had proven to give a positive impact on the development of mathematical argumentation ability of pre-service mathematics teacher, with the effect of 60.2% and the results revealed that the ability of mathematical argumentation of pre-service mathematics teacher is more developed on AQ of Climber type (21).

Although AQ studies in the field of education are rapid in Western countries, studies related to AQ, especially for future teachers in Malaysia are still lacking especially in the index or instrument development. Di (11) emphasized that the development of instruments for measuring AQ has received less attention. No universal instrument can be generalized at this time and no review of existing AQ instruments has been conducted. Besides, the agreement on the best methodological quality and measuring qualities provided by instruments has not been reached. Meanwhile, the strength of proof for each instrument is determined by methodological quality, and measurement features are largely unknown. Most of the literature also revealed that the state-of-the-art of AQ instrument development has not been explained indeed because the research only adapted the original instrument of AQ provided by Stoltz.

Recently, the literature also shows a research gap from the aspect of the AQ index development which was very limited and not been widely discussed at the Institute of Teacher Education. Several new instruments including AQ index been developed and carried out but not in IPG context such as in China which re-developed and subsequent psychometric evaluation of the Adversity Response Profile for Chinese University Students (ARP-CUS) (22). In Thailand, adversity quotient test for Grade Nine students also been developed with 40 items (10 situational) three-choice items and situational with four elements based on the theories of Stoltz and tested with classical test theory (23). Research from Indonesia at the State University of Malang (UM) also develop the AQ-based endurance dimension possessed by students through Islamic Religious Education (PAI) online learning (24). The research from (25) also provide the profile of High School students with high Adversity Quotient (AQ) in learning mathematics and revealed that high AQ students are able to face the learning of mathematics in various materials and with different models of learning. Nevertheless, all these researches are not discussed in pre-service teachers' context and the instrument development phase argument especially the model of developing the instrument also not detailed explained.

This situation further creates the need to know the AQ levels of practicum pre-service teachers through an index aimed at obtaining appropriate AQ patterns from their demographic characteristics. Therefore, it is necessary to assess the psychometric properties for the index in measuring the AQ levels of pre-service teachers. The measurement of AQ in the Malaysian context is very limited as past studies have only covered for certain aspect such as youth (14) and technical students (26) context. Based on these issues, the current study aims to develop the AQ index and test its psychometric characteristics using the modern measurement theory the Rasch model. Before the AQ index calculated, the instrument of AQ for pre-service teachers was created based on the challenges faced by the teachers while undergoing practicum training. A valid and reliable AQ index is useful in providing a variety of AQ patterns including information on the differences in the AQ levels of pre-service teachers. These differences can be seen based on demographic factors such as gender, semester of study, and field of study. Empirical evidence on AQ differences can also help stakeholders empower the right target group.

## Methodology

### Research design

The design of the study entails a survey using a quantitative approach. The type of survey used in the current study is a cross-sectional survey, where data are collected only once from a sample at a time (27). This research design is suitable because the information through the one-time data collection from the population of Bachelor Degree of Teaching Program (PISMP) in IPG pre-service teachers in the Central Zone is suitable for the construction of the index (28). The generalization of the actual research population can also be made based on the research sample (27).

### Sampling techniques

In this study, the population only entails sixth and eighth semester pre-service teachers. This is because they have completed their practicum training in the fifth and seventh semesters. Since the study comprises four phases, namely needs study, expert review study, pilot study, and validation study, different sampling techniques were used. Specifically, the needs study employed the simple sampling technique to obtain a list of challenges of the practicum pre-service teachers, while the expert review study employed the purposive sampling technique. Additionally, both the pilot study and validation study used the stratified random sampling technique because it involves the separation of the target population into different groups (29). Three strata are required in the index profile development, namely gender, semester of study, and field of study.

### Index development research phases

#### Needs study

In the needs study, a total of 105 pre-service teachers from two IPGs were involved in determining the list of challenges faced by pre-service teachers. These challenges were then combined with the CORE model for the construction of AQ index items. The two IPGs involved are IPG Ipoh Campus, Perak and IPG Campus Tun Hussein Onn, Johor. The selection of the two IPGs also represents the Northern and Southern zones. These two IPGs were chosen because they have the highest number of pre-service teachers in their respective zones. The groups of respondents required in this study include sixth and eighth semester pre-service teachers who had undergone practicum training in the previous five and seventh semesters. A simple random sampling technique was chosen in this study because this technique is compatible with descriptive studies (30). A total of 59 challenge-related items were listed with eight constructs, namely attitude, Daily lesson plan (RPH) writing, teaching materials, task load, administrative relationship, classroom management, supervision, and facilities.

#### Instrument development process

The AQ instrument was developed by combining preservice teacher challenges from need analysis with four main constructs of AQ includes Control, Ownership, Reach, and Endurance (CORE model). This instrument development model of AQ adapted by (31). There are ten steps in which are defining the construct, purpose and target of the instrument, preparing the instrument plan, developing instrument items, writing instrument implementation instructions, conducting a pilot study, conducting item analysis, performing item review and preparing the final instrument, testing the validity and reliability of instruments, and determine norms and prepare manuals. However, the last step was replaced by index development as stated by this research.

#### Expert review study

Two types of experts, namely professional experts and field experts, were selected to examine the developed items for content validity (28, 32). Nine experts comprising six professional experts and three field experts were selected. These numbers are based on the recommendation by (33) which states that six to ten experts are sufficient to evaluate constructs and items. The professional experts consist of six lecturers from Universiti Sains Islam Malaysia (USIM), Institut Pendidikan Guru Kampus Ilmu Khas (IPIK), Institut Pendidikan Guru Kampus Pendidikan Islam (IPIS), Institut Perguruan Raja Melewar (IPRM), and Pejabat Pendidikan Daerah Hulu Langat (PPDHL), while the field experts only consist of three education officers in PPDHL. The purposive sampling technique was also used in determining the selected experts. The criteria for the selection of expert's entail those who work as a teacher with more than 10 years of experience, hold a Bachelor of Education degree and still active in service.

#### Pilot study

The pilot study was conducted through item testing based on the Rasch model analysis. The main assumptions to adhere to include item compatibility, unidimensionality, and local independence, in addition to item polarity, item-individual mapping, as well as reliability and separation indexes. A total of 159 pre-service teachers from IPG Raja Melewar Campus, Negeri Sembilan were involved in the pilot study. IPG Raja Melewar Campus was selected based on the highest number of enrolments in the Southern Zone. The pilot study involved sixth and eighth semester pre-service teachers who had undergone practicum training in the last five and seven semesters. Disproportionate stratified multi-level sampling was used with three stratums were selected such as gender, semester of study, and field of study. The strata are important for showing the index patterns.

#### Validation study

The validation study involves a population of all sixth and eighth semester pre-service teachers of PISMP who took courses in IPG Central Zone. The selection of IPG Central Zone as the study population is based on the highest number of pre-service teachers in Malaysia. The enrolment of respondents in the IPG Central Zone constitutes 560 people. The sixth and eighth semester pre-service teachers of PISMP were selected based on the following considerations: (1) Pre-service teachers had taken the School-Based Learning (PBS) program in Semesters 1, 2, 3, and 4 for at least a week each semester; thus, they have received early exposure to real school situations; (2) Starting from Semesters 5, 6, and 7, these groups of pre-service teachers have undergone practicum training in schools where they get to apply theoretical and practical knowledge in a real classroom setting. Therefore, these groups of pre-service teachers should be selected as the study population as they have practiced the values of teaching professionalism, knowledge, and understanding as well as teaching and learning skills before they are sent to work in schools. The disproportionate stratified random sampling technique was selected due to the non-homogeneous populations among population members (29). The pre-service teachers were classified by gender, semester of study, and field of study. A total of 560 respondents responded but only 542 samples completed with 96.7 percent of return rate.

#### Index profile development

The calculation formula of the AQ index score adapted from the Malaysian Youth Index (IBM'16) (34) was used with percentage score calculation. A score of 100 is used as the basis to calculate the maximum score and the score of 0 serves as the minimum value. To obtain the AQ index score of the practicum pre-service teachers, the average score for all constructs was calculated. The following formula shows the calculation of the AQ score for the practicum pre-service teacher index:


$$\begin{array}{l} \text{Indicator}/\text{item score}=\sum_{I=1}\left(\frac{M_{1}-M_{s}}{R}\right)\times \;100...............\left(1\right) \end{array}$$

$$\begin{array}{l} \text{Domain}/\text{Construct score}=\left[\frac{\sum Indicator\;score}{N\;Indicator}\right]....................\left(2\right) \end{array}$$

$$\begin{array}{l} \text{Index score}=\left[\frac{\sum Domain\;score}{N\;Domain}\right].............................................\left(3\right) \end{array}$$

∑_vc_ = Score total

M_1_ = Average score

M_s_ = Minimum score

R = Range (Maximum score–Minimum score)

N = Total

To divide the AQ index level of pre-service teachers, the scale shown in Table 1 was used as adapted from Malaysian Youth Index (IBM'16) (34). Score values of 100–80 indicate a very high AQ level, followed by 79–75 (high), 74–60 (moderately high), 50–59 (moderately low), 40–49 (low), and 0–39 (very low).

**Table 1:** AQ level interpretation scale of practicum pre-service teachers.

**Score value**	**Level**	**Scale**
80–100	Very high	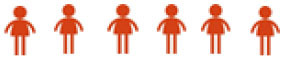
75–79	High	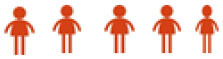
60–74	Moderately high	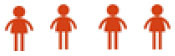
50–59	Moderately low	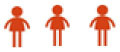
40–49	Low	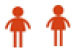
0–39	Very low	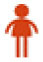

### Instrumentation

The developed AQ instrument constitutes two parts that comprise Part A, which entails the pre-service teachers' profile, and Part B, which entails the CORE model construct with control, ownership, reach, and endurance. A level of agreement with five-point Likert scale by (29) was used with 1 (strongly disagree), 2 (disagree), 3 (neutral), 4 (agree), and 5 (strongly agreed) to examine the respondents' perceptions. This non-forced choice scales with neutral point provide respondents an easy way to express their feelings (29). The pilot study was conducted on 159 respondents and the data were analyzed using Rasch analysis. Table 2 shows the items before the pilot study, which constitute 96 items and the number of final items (i.e., 54 items).

**Table 2:** Number of items—before and after pilot study.

**Before pilot study**				**After pilot study**			
**Sec**.	**Content/construct**	**No. of items**	**Total**	**Sec**.	**Content/construct**	**No. of items**	**Total**
A	Demographics	1–8	8	A	Demographics	1–5	5
B	Control	1–24	24	B	Control	1–12	12
	Ownership	25–48	24		Ownership	13–28	16
	Reach	49–72	24		Reach	29–43	16
	Endurance	73–96	24		Endurance	44–54	10
	Total number of items		96		Total number of items		54

AQ was measured using four constructs: Control (C), Ownership (O), Reach (R), and Endurance (E) or also known as CORE. Control is the most important construct in AQ. This is because control is interpreted as a defining symbol in the mind of an individual to control adverse conditions (35, 36). The control construct questions the extent to which controls are asserted (37). This construct also refers to one's degree of control over problems. The individual who obtains a high score for this construct is deemed proactive in facing challenging and able to turn difficulties into opportunities (36).

Ownership reveals the extent to which a person admits the consequences of difficulties and is willing to be held accountable for an error or failure regardless of who or what causes it (37–39). This also refers to the extent to which individuals are responsible for improving their current situation. Individuals with a high score in this construct are characterized as being responsible for their actions and learning from good results (37). Reach refers to the extent to which an individual assumes that difficulties will affect the other aspects of life (35, 37, 39). Individuals with a high score in this construct will perceive difficulties as something specific and limited as well as not affecting the other aspects of life (37, 40). Endurance is a measure of an individual's perception of the duration of adverse effects due to the difficulties that occur. Those with high endurance do not perceive difficulties as permanent; instead, they feel confident that difficulties will surely pass (37). Overall, these four components measure a person's AQ. Table 3 shows the conceptual definitions of AQ constructs.

**Table 3:** Conceptual definitions of AQ constructs (12, 37).

**Construct**	**Definition**
Control	A person's ability to handle and manage difficulties.
Ownership	A person's ability to take responsibility rather than blaming oneself when facing adversity.
Reach	A person's ability to ensure that the challenges faced do not affect his life.
Endurance	A person's ability to anticipate the duration of a challenge in his life.

### Psychometric assessment

Before obtaining the final 54 items, a total of 96 items were tested using the Rasch model. Rasch analysis involves item fit, unidimensionality, local independence, reliability, and separation index. Item fit shows the statistical fit results for the items representing each AQ construct. In this study, the fit acceptance range is between 0.77 and 1.30 logits as per (41) recommendation. A value higher than 1.30 indicates that the item is not homogeneous with other items in one measurement scale, while a value below 0.77 indicates the overlap of the construct with another item. A total of 39 items were found to misfit because the items were beyond the specified logit value, which is between 0.77 and 1.30. All four AQ constructs show a high-reliability index of items from 0.87 to 0.96. Three constructs, namely control, reach, and endurance, show the maximum values of outfit MNSQ below 1.3 logits with 1.29, 1.29, and 1.20, respectively. However, the ownership construct should be reviewed in the pilot study because its logit value exceeds 1.30. Misfit order analysis should also be done to identify items that do not fit the Rasch measurement model. The item separation index indicates that all constructs are ranging from 2.61 to 4.88. According to (42), a well-accepted value should be more than 2.0. The highest separation index was recorded by the items in the ownership construct (4.88), followed by control (4.50), endurance (3.50), and the reach construct (2.61). This suggests that the AQ items are statistically two to four times more distributed than the square root error. Item separation index can be divided into two to four strata or difficulty levels. Table 4 shows the reliability and item fit by construct.

**Table 4:** Reliability and item fit by construct.

**No**.	**Construct**	**Reliability**		***Infit*** **MNSQ**		***Outfit*** **MNSQ**	
		**Item**	**Separation**	**Max**.	**Min**.	**Max**.	**Min**.
1.	Control	0.95	4.50	1.27	0.73	1.29	0.78
2.	Ownership	0.96	4.88	1.30	0.82	1.63	0.85
3.	Reach	0.87	2.61	1.24	0.75	1.29	0.78
4.	Endurance	0.92	3.50	1.15	0.81	1.20	0.84

Table 5 shows the polarity of AQ construct items. The item polarity value of the four AQ constructs indicates the value of point measure correlation (PTMEA Corr.) or the correlation measurement point for each of the four positive constructs. Of all AQ constructs, the reach construct shows a minimum value of 0.41 for item R9 and the endurance construct shows the maximum value of item E22, which is 0.68. Overall, these findings meet the recommendation of (43), which states that a well-accepted PTMEA Corr. value is between 0.20 and 0.79. Furthermore, the polarity analysis results have statistically shown that all AQ constructs move in parallel in one direction while measuring the constructs to be measured. Subsequently, all of these items were removed from the AQ index.

**Table 5:** Polarity of AQ construct items.

**No**.	**Construct**	**PTMEA Corr**				**Total items**
		**Min**.	**Item**	**Max**.	**Item**	
1	Control	0.43	C22	0.62	C24	12
2	Ownership	0.42	O13	0.58	O9	16
3	Reach	0.41	R9	0.66	R22	16
4	Endurance	0.50	E1	0.68	E22	10
Total						54

Table 6 shows the unidimensionality of AQ construct items. In terms of item unidimensionality, Principal Component Analysis (PCA) was carried out to specify the items that measure only a single construct (14). To ensure that all items measure only a single construct, Reckase (44) recommended that the value of variance explained by measures should be more than 20 percent. Meanwhile, unexplained variance in 1st contrast that is <3.0 is deemed good, whereas the variance of the first principal component that is <5 percent is well-accepted. This indicates the existence of an obscure second dimension. In addition, the minimum variance ratio is 3:1 (43). The variance ratio is obtained by dividing the value of the variance explained by the item by the value of unexplained variance in 1st contrast (45).

**Table 6:** Unidimensionality of AQ construct items.

**No**.	**Construct**	**Variance explained by measures (%)**	**Eigen**	**Unexplained variance in 1st contrast**
1.	Control	39.8%	1.7	8.4%
2.	Ownership	38.0%	2.2	8.6%
3.	Reach	32.6%	2.0	8.6%
4.	Endurance	38.7%	1.9	11.9%
	Total	31.6%	4.0	5.0%

The variance explained by measures (%) indicates that all constructs have a value of 31.6%, which exceeds a good variance value of 20% (44). Meanwhile, unexplained variance in 1st contrast indicates that all constructs exceed the value of 3 as suggested by (45), which further indicates the absence of misfit items that tend to form a second dimension. Unexplained variance in 1st contrast also shows a value of 5.0% (46), which is generally well-accepted, while the variance ratio exceeds the minimum value of 3.9:1 (43). In terms of local independence, ten pairs of items with residual correlation standard values were recorded to range from 0.33 to 0.44, which meets the local independence requirement with a correlation value of <0.7 (43). Hence, these items do not lean with other items in the same construct. Finally, a total of 54 AQ items have been agreed for use in further studies.

The pilot study results for the examination of 96 AQ items based on the analysis using the Rasch measurement model showed 39 items that did not meet the proposed fit value, as well as 3 items that exceeded the value of 0.7 in terms of item polarity. Thus, a total of 42 items were removed from this instrument and only 54 final items were retained for the AQ measurement of pre-service teachers for the purpose of index development. The final item list of the AQ instrument is shown in Table 7. The list shows the summary of removed and retained items.

**Table 7:** Summary of removed and retained items.

**Construct**	**Number of initial items**	**Number of removed items**	**Removed item**	**Retained item**	**Total retained items**
Control	24	12	1, 2, 5, 7, 9, 13, 14, 15, 16, 17, 18, 20	3, 4, 6, 8, 10, 11, 12, 19, 21, 22, 23, 24.	12
Ownership	24	8	28, 30, 35, 36, 43, 44, 47, 48.	25, 26, 27, 29, 31, 32, 33, 34, 37, 38, 39, 40, 41, 42, 45, 46.	16
Reach	24	8	49, 52, 58, 61, 62, 65, 67, 68.	50, 51, 53, 54, 55, 56, 57, 59, 60, 63, 64, 66, 69, 70, 71, 72.	16
Endurance	24	14	74, 75, 76, 79, 80, 82, 83, 84, 85, 86, 87, 88, 90, 96.	73, 77, 78, 81,89, 91, 92, 93, 94, 95.	10
Total	96	42			54

## Results

### AQ index of pre-service teachers

The AQ index of pre-service teachers was developed based on gender (male and female), semester of study (semester six and semester eight), and program types [Arabic (BA), Malay Language (BM), Teaching English as A Second Language (TESL), Music Education (PMZ), Early Childhood Education (PRA), Islamic Education (PI), Special Education (PKHAS), Visual Arts Education (PSV), History (SEJ), Mathematics (MATE), Physical Education (PJK), Design and Technology (RBT), and Science (SN)]. Table 8 shows the AQ levels of practicum pre-service teachers in the Central Zone as a whole. 34.32% (157 people) of the pre-service teachers are at a very high level, followed by 15.50% (83 people) at a high level, 46.86% (282 people) at a moderately high level, 3.14% (19 people) at a moderately low level, and 0.19% (1 person) at a low level. However, no pre-service teachers were at a very low level. Overall, the practicum pre-service teachers have a moderately high AQ level.

**Table 8:** AQ levels of practicum pre-service teachers.

**AQ Level**	**Score scale**	**Visual scale**	**Number of pre-service teachers**	**Percentage**
Very high	80–100	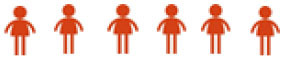	157	34.32%
High	75–79	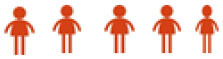	83	15.50%
Moderately high	60–74	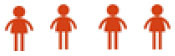	282	46.86%
Moderately low	50–59	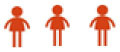	19	3.14%
Low	40–49	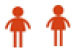	1	0.19%
Very low	0–39	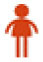	0	0%
**Total**			**542**	**100%**

**Figure 1:**
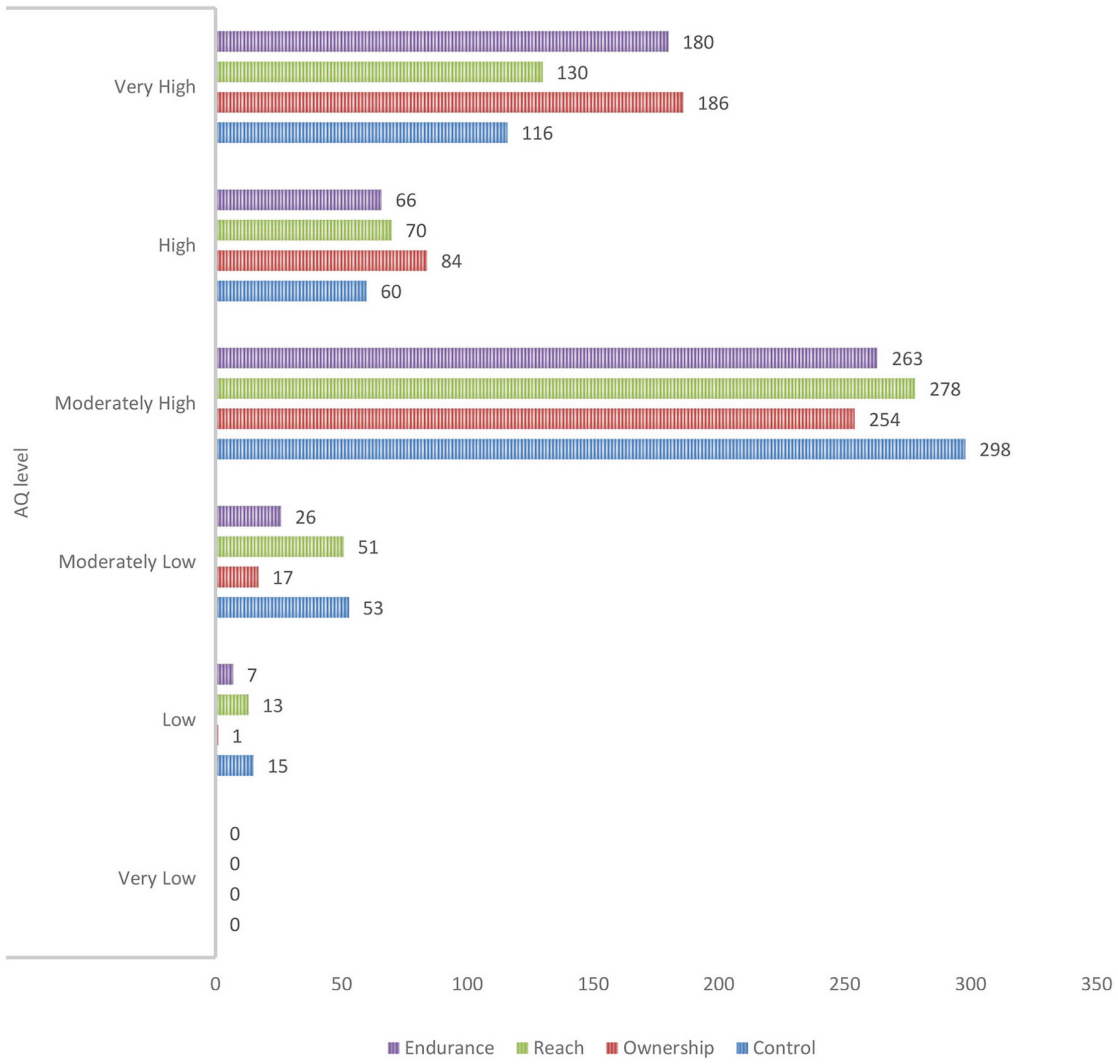
AQ levels of pre-service teachers for all the AQ constructs.

Figure 1 indicates the AQ levels of practicum pre-service teachers for the control construct. The findings show that the AQ levels of pre-service teachers are moderately high (298 people). The data distribution shows that 21.2% (116 people) of the pre-service teachers have a very high level of control, followed by 11.1% (60 people) at a high level, 55.0% (298 people) at a moderately high level, 9.8% (53 people) at a moderately low level, and 2.8% (15 people) at a moderately low level. However, no pre-service teachers have the lowest level of control. For ownership construct, the findings show that the AQ levels of pre-service teachers are moderately high (254 people). The data distribution shows that 34.3% (186 people) of the pre-service teachers have a very high level of control, followed by 15.5% (84) at a high level, 46.9% (254 people) at a moderately high level, 3.1% (17 people) at a moderately low level, and 0.2% (1 person) at a moderately low level. However, none of them has the lowest level of control.

For reach construct, the findings show that the AQ levels of pre-service teachers are moderately high (278 people). The data distribution shows that 33.2% (130) of the pre-service teachers have a very high level of control, followed by 12.2% (70 people) at a high level, 48.5% (278 people) at a moderate-high level, 4.8% (51 people) at a moderately low level, and 1.3% (13 people) at a low level. However, no pre-service teachers have the lowest level of control. For endurance construct, the findings that the AQ levels of the pre-service teachers are moderately high (263 people). The data distribution shows that 33.2% (180 people) of the pre-service teachers have a very high level of control, followed by 12.2% (66 people) at a high level, 48.5% (263 people) at a moderately high level, 4.8% (26 people) at a moderately low level, and 1.3% (7 people) at a moderately low level. However, none of the pre-service teachers has the lowest level of control. The explanation also stated in Table 9 which mention the crosstab between AQ levels and constructs.

**Table 9:** Crosstab between AQ levels and constructs (*n* = 542).

**AQ Construct**	**AQ Level**					
	**Very high**	**High**	**Moderately high**	**Moderately low**	**Low**	**Very low**
	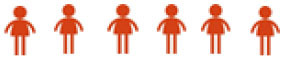	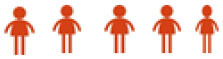	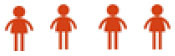	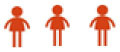	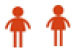	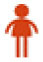
Control	116 (21.4%)	60 (11.1%)	298 (55.0%)	53 (9.8%)	15 (2.8%)	0 (0%)
Ownership	186 (34.3%)	84 (15.5%)	254 (46.9%)	17 (3.1%)	1 (0.2%)	0 (0%)
Reach	130 (33.2%)	70 (12.2%)	278 (48.5%)	51 (4.8%)	13 (1.3%)	0 (0%)
Endurance	180 (33.2%)	66 (12.2%)	263 (48.5%)	26 (4.8%)	7 (1.3%)	0 (0%)

### AQ index profile of practicum pre-service teachers by construct

**Figure 2:**
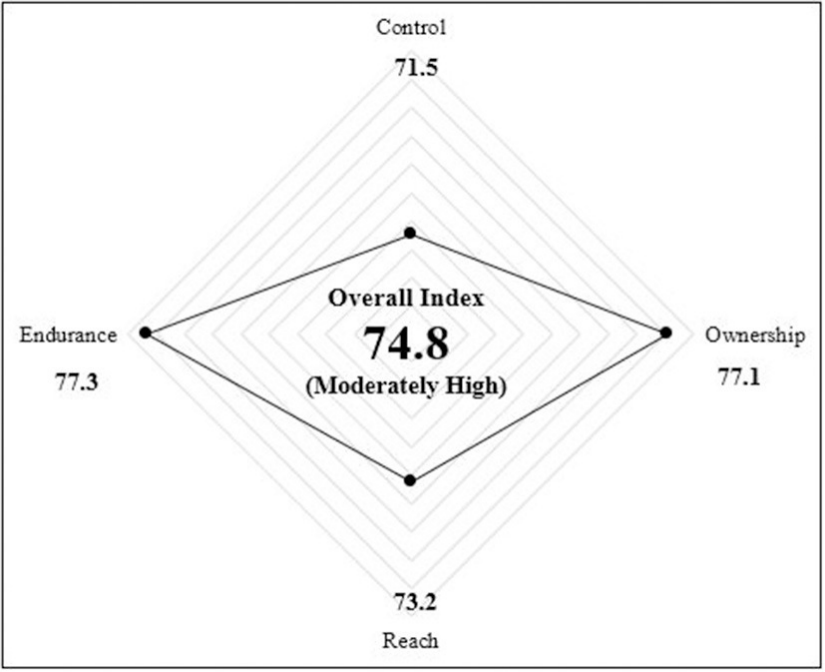
Overall AQ index score.

Table 10 show the overall index score of the AQ of practicum pre-service teachers with moderately high level of a score value of 74.8. The two constructs that are at a high level is endurance (score of 77.3) and ownership (score of 77.1). Meanwhile, the other two constructs show a moderately high level: reach (score of 73.2) and control (score of 71.5). Figure 2 also shows the overall AQ Index Score for all the construct visually.

**Table 10:** Overall AQ index scores.

**Construct**	**Score**	**Level**	**Level**
Control	71.5	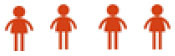	Moderately high
Ownership	77.1	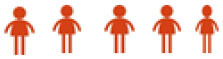	High
Reach	73.2	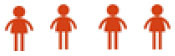	Moderately high
Endurance	77.3	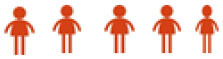	High
Pre-service teacher AQ index score	74.8	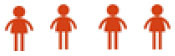	Moderately high

### AQ profile of pre-service teachers based on gender

The AQ index for each construct based on gender is shown in Table 11. The AQ index value of male practicum pre-service teachers is higher with a score of 76.29 (high) than the female practicum pre-service teachers with a score of 74.09 (moderately high).

**Table 11:** AQ index values of practicum pre-service teachers by gender.

**Gender**	***n***	**Control**	**Ownership**	**Reach**	**Endurance**	**AQ**	**Level**
Male	176	74.36	77.53	74.90	78.37	76.29	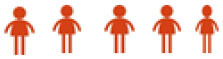
Female	366	70.19	76.90	72.39	76.86	74.09	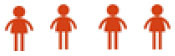

**Figure 3:**
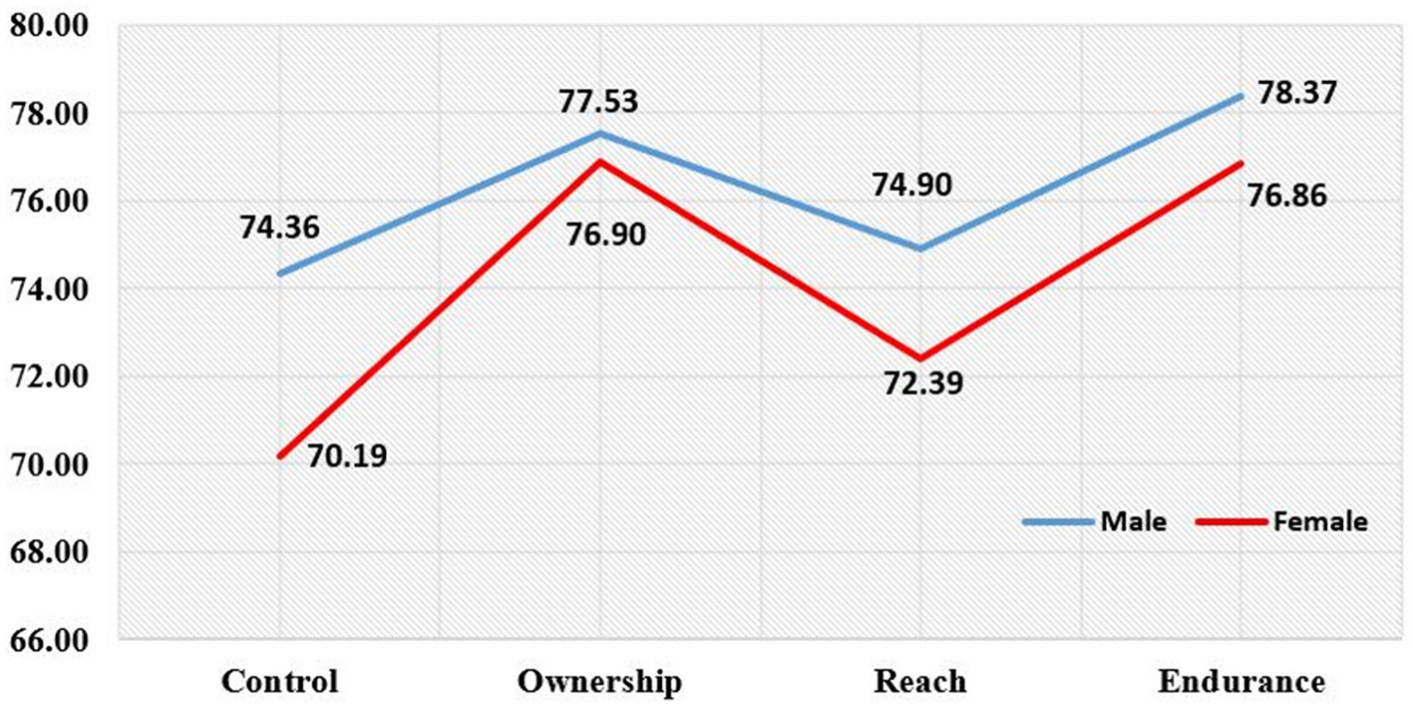
AQ index by construct based on gender.

Figure 3 show the AQ index by construct based on gender. For the control construct, the index score of male pre-service teachers is higher (74.36) than females (70.19). For the ownership construct, the index score of male pre-service teachers is higher (77.53) than that of female pre-service teachers (76.90). For the reach construct, male pre-service teachers recorded a higher index score (74.70) than the females (72.39). Finally, for the endurance construct, the male pre-service teachers' index score is above (78.37) the index score of the female pre-service teachers (76.86).

### AQ profile of pre-service teachers based on semester of study

Table 12 shows the AQ index value of pre-service teachers as a whole based on their semester of study. The overall findings show that the index score of the eighth semester pre-service teachers is higher (score of 75.28) than the sixth semester pre-service teachers (score of 74.38).

**Table 12:** AQ index values of practicum pre-service teachers by semester of study.

**Semester**	***n***	**Control**	**Ownership**	**Reach**	**Endurance**	**AQ**	**Level**
Six	291	71.13	77.22	72.58	76.58	74.38	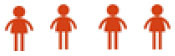
Eight	251	71.95	76.98	73.94	78.24	75.28	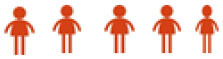

**Figure 4:**
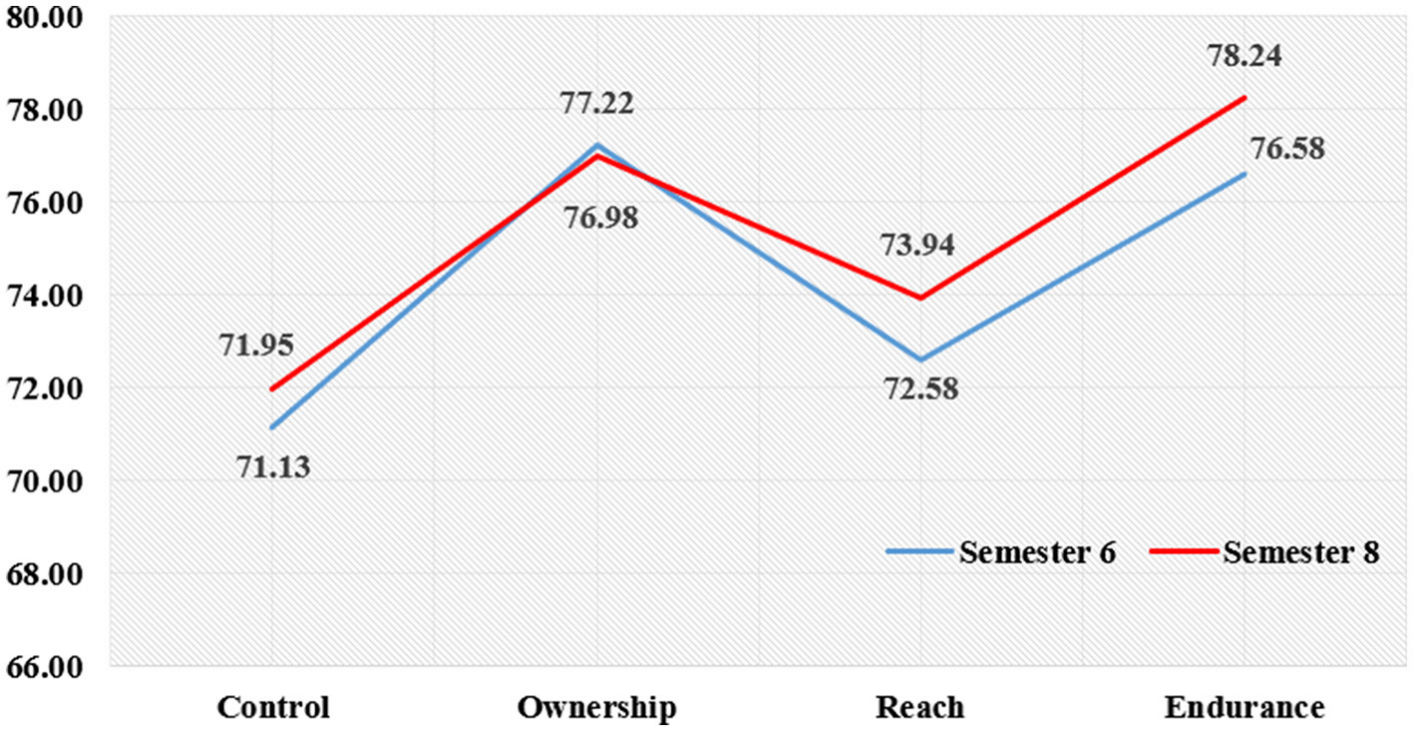
AQ index by construct based on semester of study.

Figure 4 show the AQ index for pre-service teachers based on semester of study. The endurance construct (77.41) is dominated by the sixth semester pre-service teachers (76.58) and eighth semester pre-service teachers (78.24). This is followed by the ownership construct (77.10) as the score obtained by the sixth semester pre-service teachers (77.22) exceeds that of the eighth semester pre-service teachers (76.98). In the reach construct (77.41), the score obtained by the eighth semester pre-service teachers (73.94) outperforms the sixth semester pre-service teachers (72.58). Finally, the control construct is the least dominant (71.54). Overall, the eighth semester pre-service teachers (71.54) outperform the sixth semester pre-service teachers (71.95).

### AQ profile of pre-service teachers based on field of study

Table 13 showed that the overall index score obtained by SN pre-service teachers is the highest (80.45), while the PSV pre-service teachers obtained the lowest index score (70.29).

**Table 13:** AQ profile of pre-service teachers based on field of study.

**Field**	***n***	**Control**	**Ownership**	**Reach**	**Endurance**	**AQ index**	**Level**
BA	45	73.84	80.49	74.03	81.06	77.35	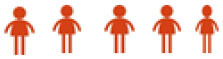
BM	52	72.16	78.55	74.43	79.38	76.13	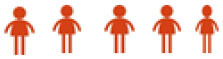
TESL	109	67.97	74.70	70.81	75.30	72.19	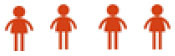
PMZ	30	73.75	78.80	75.94	78.50	76.75	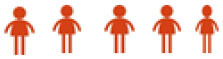
PRA	9	73.38	84.72	74.83	82.78	78.93	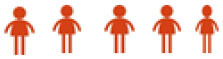
PI	68	75.34	79.73	75.11	79.01	77.30	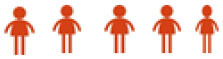
PKHAS	74	73.34	75.42	73.29	77.16	74.80	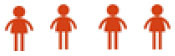
PSV	20	67.92	73.67	70.31	69.25	70.29	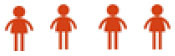
SEJ	7	75.46	81.68	79.51	81.53	79.55	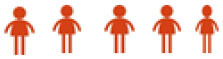
MATE	25	68.99	78.67	73.14	77.40	74.55	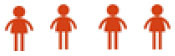
PJK	36	72.34	73.26	70.88	74.72	72.80	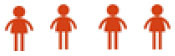
RBT	49	67.26	75.48	71.49	75.56	72.45	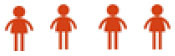
SN	18	76.74	81.51	80.21	83.33	80.45	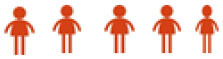

Arabic (BA), Malay Language (BM), Teaching English as A Second Language (TESL), Music Education (PMZ), Early Childhood Education (PRA), Islamic Education (PI), Special Education (PKHAS), Visual Arts Education (PSV), History (SEJ), Mathematics (MATE), Physical Education (PJK), Design and Technology (RBT), and Science (SN).

**Figure 5:**
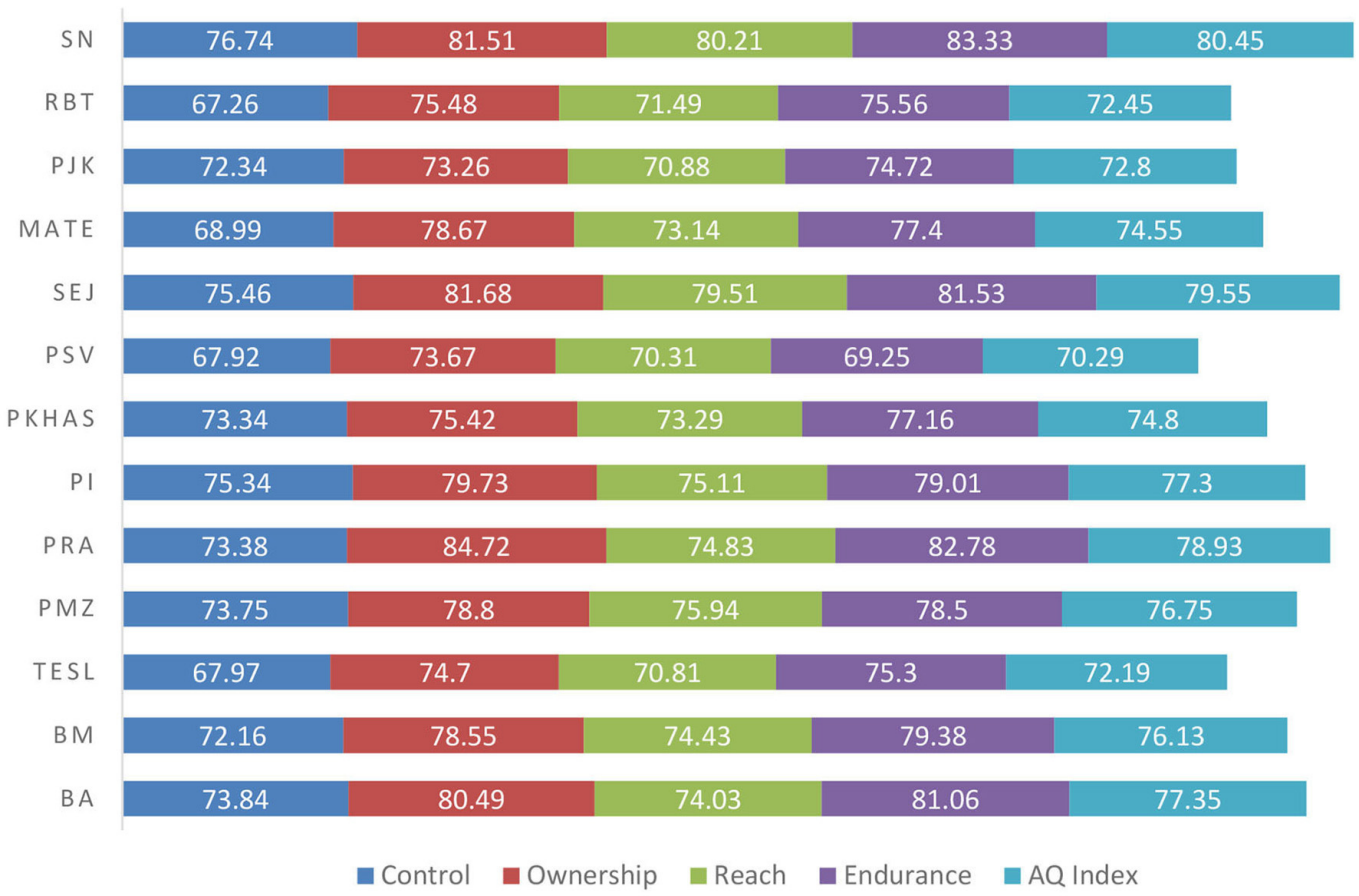
AQ index comparison of pre-service teachers based on field of study. Arabic (BA), Malay Language (BM), Teaching English as A Second Language (TESL), Music Education (PMZ), Early Childhood Education (PRA), Islamic Education (PI), Special Education (PKHAS), Visual Arts Education (PSV), History (SEJ), Mathematics (MATE), Physical Education (PJK), Design and Technology (RBT), and Science (SN).

The findings for the control construct show that SN recorded the highest level of control (81.51), while the RBT shows the lowest index score (67.26). Meanwhile, the index scores for TESL (67.97), PSV (67.92), MATE (68.99) and RBT (67.26) are all below the overall index score of the control construct (71.5). The ownership construct show PRA with the highest score (84.72), while PJK recorded the lowest score (score 73.26). Meanwhile, five fields of studies show a score below the overall score of the ownership construct (77.10): TESL (74.70), PKHAS (75.42), PSV (73.67), PJK (73.26), and RBT (75.48). The findings for the reach construct show SN with the highest score (80.21), while PSV recorded the lowest score (70.31). Nonetheless, TESL (70.81), PSV (70.31), MATE (73.14), PJK (70.88), and RBT (71.49) recorded a score below the overall score of the reach construct (73.20). For endurance, SN recorded the highest score with 83.33, while PSV recorded the lowest score with 69.25. However, six fields of study are below the overall index score of the endurance construct (77.30) namely TESL (75.30), PKHAS (77.16), PSV (69.25), PJK (74.72), and RBT (75.56). Based on constructs, the PSV, TESL and RBT respondents AQ index are below average. However, the index of BA, BM, PMZ, PRA, PI, and SEJ respondents were more than average. Figure 5 shows that the highest overall index of AQ was SN, followed by SEJ and PRA. The bottom rank were PSV, TESL and RBT.

## Discussion

The findings have shown that the overall AQ level of pre-service teachers is moderately high. Two constructs (endurance and ownership) are at a high level, while the other two constructs (reach and control) are at a moderately high level. The AQ construct that recorded the highest score is endurance, followed by the ownership, reach, and control constructs. This shows that the pre-service teachers have good self-endurance and are willing to work hard despite the difficult and challenging tasks; besides, the pre-service teachers can also easily establish good relations with the school and students. Sing et al. (47) stated that the self-esteem, motivation, fighting spirit, creativity, sincerity, positive attitude, optimism, and good emotional health are all characteristics of someone with high AQ.

In Malaysia, the are almost no research of AQ on the pre-service teacher including the index development for that purpose. The research exist only for technical students from the study that showing moderate AQ findings (48, 49). However, Indonesia was different when most of the recent research of AQ are focusing on pre-service mathematics teacher. These past researches conducted not only limited during their internship, but also conducted at the end or after their internship. The findings from (25) had analyzed the profile of High School students with high Adversity Quotient (AQ) in learning mathematics show that students with high AQ able to face the learning of mathematics in various materials and with different models of learning. Study by (20) also proven that AQ gives positive influence to the development of mathematical understanding ability of pre-service mathematics teacher with the influence of 57.3 percent. Result from (21) mentioned that AQ gives a positive impact on the development of mathematical argumentation ability of pre-service mathematics teacher, with the effect of 60.2 percent. Furthermore, the ability of mathematical argumentation of pre-service mathematics teacher is more developed on AQ of climber. This revealed that AQ had a potential to helps the teachers and students improving the session for teaching and learning for mathematics.

The pre-service teachers were able to obtain a moderately high AQ score owing to their self-improvement training. The *Bina Insan Guru* Program (Teachers' Human Development Programme), which is compulsory for all pre-service teachers includes an element of AQ, namely intelligence in facing challenges. Other factors that may contribute to the high AQ level of pre-service teachers also involve curriculum and co-curricular aspects. Various approaches and strategies of student-centered learning have allowed pre-service teachers to be cooperative and collaborative among themselves. In terms of co-curriculum, activities such as camping, outdoor education, clubs, associations, and uniformed units also provide opportunities for pre-service teachers to hone their leadership, resilience, and motivational characteristics. The pre-service teachers' involvement in these programs and activities has successfully inculcated the qualities of self-awareness, self-control, motivation, and social skills among them and subsequently managed to increase their AQ levels. Yazon (50) agreed and found that pre teachers should continue to do what they enjoy and love because success is much easier when you are passionate about what you are doing, regardless of whether you have done it yourself. It is critical to cultivate a passion for something or to focus on doing what you enjoy.

Hema and Gupta (51) stated that teachers should have the resilience to face student challenges at school. Teachers need to build good relationships with other fellow teachers, school staff, parents, and society. Thus, educational institutions must produce human resources and potential educators who are knowledgeable, competent, able to think and solve problems, and have a high AQ in facing challenges. A high AQ score can not only reduce negative emotions but also reduce boredom and despair. The findings of this study are in line with the resiliency theory by Grotberg (52) where a person who can rise from pain is deemed successful and able to control emotions well. From the point of view of AQ constructs, a person with the ability to control challenges in learning is resilient and not easy to admit defeat. If the pre-service teachers are able to think well, they will understand the root causes of challenges and subsequently find solutions (53).

### AQ index of pre-service teachers based on gender

The findings have shown differences in AQ levels between male pre-service teachers and female pre-service teachers where the males have higher AQ levels than the females. These findings are in line with previous studies such as Hema and Gupta (51) that examines pre-service teachers, the AQ levels of male pre-service teachers were higher than female students from the problem-solving aspect. The findings were also supported by Ahmad Zamri and Syed Mohamad (49) in their studies involving USM students, which found that male students were more resilient than female students. The findings are also supported by the contemporary theory, which portrays men as competitors and committed to a task, especially from an employment point of view. On the other hand, females are more dominant in terms of empathy, particularly interpersonal.

However, the findings reported by (48, 54) show otherwise. The AQ level of female students was higher than that of male students and the female students were also more motivated and concerned in carrying out their responsibilities than male students. This finding was supported by Wiwin and Latifah (55) who examined students in Aceh, Indonesia and showed that female students were more responsible and good at controlling challenging situations. Female students were also good at organizing assignments; ultimately, their difficulties can be overcome well. Meanwhile, Ahmad Zamri and Syed Mohamad (49) in the context of USM specifically found that female students are competent in management and have better control than men. Overall, the findings of this study have shown that the AQ levels of male pre-service teachers are higher than female pre-service teachers in all constructs (control, ownership, reach, and endurance). This is strengthened by the aggressive, assertive, prominent, competitive, dominant, coercive, and more independent male nature. Meanwhile, female nature is said to be gentle, affectionate, patient, and dependent on others (56). Kiger (57) revealed that these differences are important in showing how men and women respond to difficulties. Women are more likely to blame themselves when they encounter failure and assume that the failure will last forever. On the other hand, men are more likely to characterize failure as something temporary (58, 59).

### AQ index of pre-service teachers based on semester of study

The findings have shown differences in AQ levels between sixth semester pre-service teachers and eighth semester pre-service teachers. The eighth semester pre-service teachers have higher AQ levels than the sixth semester pre-service teachers, and the eighth semester pre-service teachers also recorded higher scores than the sixth semester pre-service teachers in the control, reach, and endurance constructs. However, the score for the reach construct was found to be declining by the final semester.

The findings of this study are in line with a study conducted by Huijuan (38) which found that final year students have higher AQ levels. This is because the students are more mature and have faced challenges before. Likewise, Macasaet (39) found similar findings where senior students recorded high AQ levels and were better than junior students. However, these findings contradict past studies that demonstrated higher AQ levels of new students than final year students (49, 60). Meanwhile, Mohd Effendi's (48) study in the context of polytechnic students found that first-year students have higher AQ levels than second- and third-year students. First-year students tend to have more challenges in the transition to a new environment from secondary education to higher education. They also tend to face various challenges such as adaptation to the new atmosphere, communication, self-reliance, and financial affairs (12). The study by Shen (59) involves two groups of teachers, i.e., senior and new teachers in Taiwan, where new teachers were found to have higher AQ levels than senior teachers. This is due to the enthusiasm of new teachers who have just been involved in education compared to senior teachers who are becoming less enthusiastic because they have been in the field for a long time (12).

The current study shows that the AQ levels of the eighth semester pre-service teachers are higher than the sixth semester group. These results show an increase in AQ as age increases, which means that AQ can be improved through experience (12). This is because the eighth semester pre-service teachers are more mature in facing the challenges of conducting practicum training in schools. Furthermore, this is their second practicum series. They first gained the experience of becoming teachers in schools and became more and more aware of all the challenges and obstacles (61). On the other hand, this is their first experience of practicum practice in schools for the sixth semester group. Those who are in the eighth semester could control and manage stress as well as be able to solve the problems encountered due to their training experience (62).

Theoretically, the findings of this study are in line with the CORE model by Stoltz (12) in which a person's response pattern to a challenge or difficulty is the result of repeated learning. An individual will learn from repeated failures and subsequently respond to those failures; eventually, this becomes a pattern of individual reactions (12, 63). These findings are also supported by Macaseat's (39), which explains that a person's age affects how they control difficulties. Young pre-service teachers (semester six) have less ability than those who are more mature (semester eight) in handling emotions. Young pre-service teachers also have fewer effective strategies to overcome this weakness because younger individuals do not have much life experience, are independent, and have less developed personal identities than those who are older (12).

### AQ index of pre-service teachers based on field of study

In terms of field of study, the findings have shown that SN recorded a very high AQ score, while PSV recorded the lowest AQ level. This clearly shows that all fields of study have different levels of AQ.

The findings of this study are in line with the findings of Cura and Gozum (64), which show the differences in AQ levels among fields of study. The study program that recorded the highest AQ is Computer Engineering, while Computer Studies Major in Information Technology recorded the lowest AQ level. The findings also support the study by Huijuan (38), which showed differences in the AQ levels of students in Nursing, Psychology, Media Communication, and Business Administration programs. The study also found that students from the Psychology program recorded a high AQ level and students in the Nursing program recorded the lowest AQ level. The same goes for the findings reported by Yazon and Manaig (61) on the AQ levels of 126 students in the Philippines, in which science and mathematics students recorded high AQ levels compared to other fields of study.

Studies related to AQ levels based on the field of study in the context of education are very rare because, despite the different fields, the challenges that students receive are not extensively different (53). However, the findings of this study prove the opposite where science pre-service teachers showed higher AQ levels and dominated the constructs of control, reach, and endurance compared to other fields of study. This is evidenced by Halpern et al. (65), which found that students in the field of science have a high level of intelligence compared to students in the field of literature. This is because Science students have a more realistic nature as they are guided by theory, physical, and proof. Science students are also analytical, rational-minded, and have logical nature. On the other hand, literature students are more creative, imaginative, and emotional (31). Furthermore, in the classroom, science pre-service teachers encourage students to develop questions that contribute to an output to avoid unproductive discussions (66).

As for the ownership construct, PRA pre-service teachers were found to achieve a very high level compared to other fields. This is because PRA pre-service teachers are trained to educate children aged 4–6 years in terms of the children's preparation before entering real schools (67). This role is assumed by PRA pre-service teachers to ensure that PRA students master basic education; thus, the teachers are more responsible and ready to face any challenges. Besides, 2–6 year-olds tend to be influenced by their surroundings such as parents, friends, and teachers (67). To ensure the success of these preschoolers, PRA pre-service teachers are more optimistic and do not give up easily. This is in line with the Theory of Optimism by Seligman (68, 69), which states that optimistic individuals will be more successful than pessimistic individuals.

In conclusion, empirical evidence related to AQ differences in terms of field of study is very useful in understanding the AQ patterns of pre-service teachers. Any fields of study that comprise teachers with low AQ levels are certainly not able to highlight quality work. This is because the teachers cannot afford to turn challenges into success. Furthermore, pre-service teachers who recorded low AQ levels in terms of field of study should also be given more attention, for example, by giving more exposure to AQ aspects through activities such as lectures, workshops, group activities, and even training approaches. The pre-service teachers should also be guided to practice AQ in daily life since AQ is the main determinant in the formation of high-quality teachers (12).

## Limitations to the study

Due to practical constraints, this paper cannot provide a comprehensive review of certain aspects due to limitations. First, the data were initiated only from the Institute of Teacher Education (IPG) in the Central Zone, Malaysia and the results may not be fully valid for other teacher education training and zones. Second, the data were only collected from self-reported questionnaires; hence, this study should implement a variety of approaches such as a qualitative or mixed-method. Thirdly, the AQ Index only for the pre-service teachers and cannot be generalized to other samples. Finally, the AQ scores were only distinguished by gender, semester of study, and field of study. Thus, more research on this topic needs to be undertaken other than this demographic scope for a better understanding of AQ.

## Conclusions

This article aims to develop the Adversity Quotient (AQ) index of pre-service teachers in the Institute of Teacher Education (IPG). The index shows that the AQ of pre-service teachers is at a moderately high level. Specifically, the highest score entails the construct of endurance, followed by ownership, reach, and control. The findings of this study have also shown that the AQ index of male pre-service teachers is higher than that of females. In addition, pre-service teachers in the final semester have a higher AQ index than pre-service teachers of earlier semesters. Science (SN) pre-service teachers also recorded very high AQ index scores, while Visual Arts Education (PSV) recorded the lowest AQ level. In summary, these results had a good psychometric assessment using the Rasch model.

The findings of this study have several important implications for future practice such as theoretical implications for the CORE model with the development of CORE conceptualization in the context of pre-service teachers. In addition, the items developed in this study are based on challenges in the context of IPG pre-service teachers and were adapted to the CORE model. There are also implications for IPG as the findings of this study have given ideas to change the direction of the program to improve the AQ intelligence of pre-service teachers in all courses, workshops, and lectures. The same goes for the implications of the study for the Ministry of Education Malaysia as a government body that is directly involved in drafting the education curriculum to consider the intelligence element of AQ in the new teacher training curriculum in IPG. Another important practical implication in terms of measurement is that the construction of the AQ index in this study has the potential to strengthen the screening process of teacher selection to strengthen the existing psychometric test findings.

The scope of this study was limited to certain area such as the sample only covered IPG and the Central Zone only. The challenges also restricted to 59 challenges were listed with eight constructs. Future studies will have recommended some ideas in order to cater this limitation. First, the sample of the study should be expanded to the context of pre-service teachers in the IPG of other zones such as the Southern, Northern, Eastern, and Borneo Zones rather than only the Central Zone. In addition, further studies can be conducted on pre-service teachers at Public Universities (UA) and Private Higher Educational Institutions (IPTS). Institutional differences are determined by different challenges compared to IPGs; thus, more new findings will be found by future researchers. Besides, further studies can be done qualitatively as well as by using a mixed-method rather than using questionnaires alone. Further exploration can also be done to catalyze ideas for the intervention process to strengthen the AQ of IPG pre-service teachers more effectively.

## Data availability statement

The original contributions presented in this study are included in the article/Supplementary material, further inquiries can be directed to the corresponding author.

## Ethics statement

The studies involving human participants were reviewed and approved by Bahagian Perancangan dan Penyelidikan Dasar Pendidikan (Education Planning and Research Division), Ministry of Education Malaysia. Written informed consent for participation was not required for this study in accordance with the national legislation and the institutional requirements.

## Author contributions

RMA and MEEMM: conceptualization, validation, resources, and data curation. RMA: methodology, software, formal analysis, investigation, writing—original draft preparation, and project administration. MEEMM: writing—review and editing, visualization, supervision, and funding acquisition. Both authors have read and agreed to the published version of the manuscript.

## Funding

This study was funded by the Faculty of Education, Universiti Kebangsaan Malaysia (UKM) with a Publication Reward Grant (GP-2021-K021854) and the Ministry of Higher Education (MOHE), Malaysia through the Fundamental Research Grant Scheme (FRGS) (FRGS/1/2018/SSI09/UKM/02/1).

## Conflict of interest

The authors declare that the research was conducted in the absence of any commercial or financial relationships that could be construed as a potential conflict of interest.

## Publisher's note

All claims expressed in this article are solely those of the authors and do not necessarily represent those of their affiliated organizations, or those of the publisher, the editors and the reviewers. Any product that may be evaluated in this article, or claim that may be made by its manufacturer, is not guaranteed or endorsed by the publisher.

## References

[1] Osman BHA Basar MN. Amalan Pengajaran dan Pembelajaran Abad Ke-21 dalam Kalangan Pensyarah Institut Pendidikan Guru Kampus Ipoh. J Penyelid Dedik. (2016) 10:1–25. Retrieved from: https://sme6044.files.wordpress.com/2017/03/abad-21-ipg-ipoh.pdf

[2] Napanoy JB Gayagay GC Tuazon JRC. Difficulties encountered by pre-service teachers: basis of a pre-service training program. Univers J Educ Res. (2021) 9:342–9. 10.13189/ujer.2021.090210

[3] Walshe N Driver P. Developing reflective trainee teacher practice with 360-degree video. Teach Teach Educ. (2019) 78:97–105. 10.1016/j.tate.2018.11.009

[4] Foncha JW Abongdia JFA Adu EO. Challenges encountered by student teachers in teaching english language during teaching practice in East London, South Africa. Int J Educ Sci. (2015) 9:127–34. 10.1080/09751122.2015.11890302

[5] Diala H Ibrahim AS Yousef A Ferial AA. Student-teachers' perspectives of practicum practices and challenges. Eur Sci J. (2014) 10:191–214. 10.19044/esj.2014.v10n13p%p31612549

[6] Md Dazali NS Awang I. Tahap Keyakinan Diri dalam Kalangan Pelajar Sarjana Muda Pendidikan, di Universiti Utara Malaysia. Educ J Soc Sci. (2017) 3:30–40. 10.37134/ejoss.vol3.1.4.2017

[7] Malik A Siraj S Abdullah MRTL AsraSakika K. Keperluan Dan Penerimaan Guru Pelatih Terhadap Pelaksanaan M-Pembelajaran Dalam Pendidikan Guru. J Kurikulum Pengajaran Asia Pasifik. (2017) 5:62–70. Retrieved from: https://ejournal.um.edu.my/index.php/JUKU/article/view/8228/5698

[8] Meerow S Newell JP Stults M. Defining urban resilience: a review. Landsc Urban Plan [Internet]. (2016) 147:38–49. 10.1016/j.landurbplan.2015.11.01130301209

[9] Serliana A Utami PP Kamil AB. Pre-service teachers' challenges in classroom management during teaching practice. J Lang Teach Learn Linguist Lit. (2021) 9:73–80. 10.24256/ideas.v9i2.2075

[10] Wang Y. Building teachers' resilience: practical applications for teacher education of China. Front Psychol. (2021) 12:1–5. 10.3389/fpsyg.2021.738606 34456836 PMC8397577

[11] Di Y. A critical review of adversity quotient instruments: using the cosmin checklist. High Educ Orient Stud. (2021) 1:35–44. 10.54435/heos.v1i4.31

[12] Stoltz PG. Adversity quotient: turning obstacles into opportunities. Hoboken, NJ: John Wiley and Sons (1997).

[13] Matore MEEM Khairani AZ Razak NA. Development and psychometric properties of the adversity quotient scale : an analysis using rasch model and confirmatory factor analysis. Rev Argentina Clí*nica Psicológica*. (2020) 2018:574–91. 10.24205/03276716.2020.105535681973

[14] Matore MEEM Zainal MA Mohd Noh MF Khairani AZ Abd Razak N. The development and psychometric assessment of malaysian youth adversity quotient instrument (MY-AQi) by combining rasch model and confirmatory factor analysis. IEEE Access. (2021) 9:13314–29. 10.1109/ACCESS.2021.3050311

[15] Nikam VB Uplane MM Adversity Adversity quotient and defense mechanism of secondary school students. Univers J Educ Res. (2013) 1:303–308. 10.13189/ujer.2013.010405

[16] Koray M. Analysis of the problems posed by pre-service primary school teachers in terms of type, cognitive structure and content knowledge. Int J Educ Methodol. (2019) 5:577–90. 10.12973/ijem.5.4.577

[17] Eren A Rakicioglu-Söylemez A. Pre-service teachers' professional commitment, sense of efficacy, and perceptions of unethical teacher behaviours. Aust Educ Res. (2021) 48:337–57. 10.1007/s13384-020-00396-7

[18] Havia J Lutovac S Komulainen T Kaasila R. Preservice subject teachers ' lack of interest in their minor subject: is it a preservice subject teachers ' lack of interest in their minor subject: is it a problem? Int J Sci Math Educ. (2022) 1:1–19. 10.1007/s10763-022-10277-3

[19] Muthamizhselvan M Kumar ACLA. study of spiritual intelligence among graduating students. Asian J Multidimens Res. (2019) 8:91. 10.5958/2278-4853.2019.00087.9

[20] Hidayat W Husnussalam H. The adversity quotient and mathematical understanding ability of pre- service mathematics teacher. In: Journal of Physics: Conference Series. Bristol: IOP Publishing (2019).

[21] Hidayat W Wahyudin Prabawanto S. The mathematical argumentation ability and adversity quotient (AQ) of pre-service mathematics teacher. J Math Educ. (2018) 9:239–48. 10.22342/jme.9.2.5385.239-248

[22] Wang X Yan Z Huang Y Tang A Chen J. Re-developing the adversity response profile for Chinese University Students. Int J Environ Res Public Health. (2022) 19:6389. 10.3390/ijerph19116389 35681973 PMC9180553

[23] Juntadit P. The Development Of Adversity Quotient Test For Graded 9 Students. Watthana: Srinakharinwirot University (2020).

[24] Mardiana D. Adversity quotient and the development of students ' endurance dimensions in the new normal era : a study of islamic religious education online learning at the State University of Malang. Intelekt J Pendidik dan Stud Keislam. (2022) 12:19–33. 10.33367/ji.v12i1.2278

[25] Hidayat W Prabawanto S. Student profile with high adversity quotient in math learning Student profile with high adversity quotient in math learning. In*: Journal of Physics: Conf Series*. Bristol: IOP Publishing (2018). p. 1–6.

[26] Matore MEEM Hapiz NM Al Matore ERM. The characteristics of quitters, campers and climbers of adversity quotient (AQ) on polytechnic students from gender perspectives. In:Abdollah MF Bin Amiruddin H Singh ASP, editors. Proceedings of Mechanical Engineering Research Day. Melaka: Universiti Teknikal Malaysia Melaka (2020). p. 257–8.

[27] Creswell JW. Educational Research: Planning, Conducting, and Evaluating Quantitative and Qualitative Research. 6th ed. Boston: Pearson Education (2018).

[28] Gay LR Mills GE. Educational Research: Competencies for Analysis and Applications. 12th ed. Upper Saddle River, NJ: Merrill Prentice Hall (2018).

[29] Hair JFCelsi MW Harrison DE. Essentials of Marketing Research. 5th ed. New York, NY: McGraw-Hill Education (2020).

[30] Sekaran U Bougie R. Research Methods for Business: A Skill Building Approach. 8th ed. New Delhi: John Wiley and Sons (2020).

[31] Miller LA Lovler RL. Foundations of Psychological Testing: A Practical Approach. 6th ed. California: SAGE Publications Inc (2019).

[32] Rubio DM Berg-Weger MS Tebb S Lee ES Rauch S. Objectifying content validity: conducting a content validity study in social work research. Soc Work Res. (2003) 27:94–105. 10.1093/swr/27.2.94

[33] Terwee CB Prinsen CAC Chiarotto A Westerman MJ Patrick DL Alonso J. COSMIN methodology for evaluating the content validity of patient-reported outcome measures: a Delphi study. Qual Life Res. (2018) 27:1159–70. 10.1007/s11136-018-1829-0 29550964 PMC5891557

[34] Institut Penyelidikan Pembangunan Belia Malaysia. Penilaian Outcome Indeks Belia Malaysia. (IBM '16) Mengukur Tahap Kualiti and Kesejahteraan Hidup Belia Malaysia. Putrajaya: Institut Penyelidikan Pembangunan Belia Malaysia (2016).

[35] Bandura A. Social Learning Theory. New Jersey, NJ: Prentice Hall (1997). Retrieved from: https://www.proquest.com/docview/1518876511?pq-origsite=gscholar&fromopenview=true

[36] Paramanandam P Shwetha R. Adversity Quotient (AQ) as a predictor of job satisfaction. Int J Glob Bus Manag Res. (2013) 1:27–37.

[37] Stoltz PG Weihenmayer E. The Adversity Advantage: Turning Everyday Struggles Into Everyday Greatness. 2nd ed. New York, NY: Fireside (2010).

[38] HuiJuan Z. The Adversity Quotient and Academic Performance Among College Students at St. Joseph's College, Quezon City. Bachelor Thesis. New York: St. Joseph's College (2009).

[39] Macasaet CJA Cornista GAL Lipa AY. Adversity Quotient ^®^ *and Achievement Motivation of Selected Third Year and Fourth Year Psychology Students of De La Salle*. Bachelor Thesis. Batangas: De La Salle Lipa (2013).

[40] Pasaribu ACR. Hubungan antara self esteem dan adversity intelligence suatu studi pada mahasiswa Universitas HKBP Nommensen Medan. VISI. (2011) 19:399–416. Retrieved from: http://akademik.uhn.ac.id/portal/public_html/MM/VISI-UHN/2011/VISI_Vol_19_No_1-2011/3_ASINACRISTINA.doc

[41] Fisher JWP. Rating scale instrument quality criteria. Rasch Meas Trans. (2007) 21:1095. Retrieved from: http://www.rasch.org/rmt/rmt211a.htm

[42] Bond TG FoxCM. Applying the Rasch Model: Fundamental Measurement in the Human Sciences. New Jersey, NJ: Routledge (2015).

[43] Linacre JM. A User's Guide to WINSTEPS: Rasch Model Computer Programs. Chicago: MESA Press (2012).

[44] Reckase D. Unifactor latent trait models applied to multifactor tests: Results and implications. J Educ Stat. (1979) 4:207–30. 10.3102/10769986004003207

[45] Linacre J. M. (2009). A user's guide to WINSTEPS: Rasch Model Computer Programs. Chicago: MESA Press.

[46] Linacre JM. A User's Guide to WINSTEPS: Rasch Model Computer Programs. Chicago: MESA Press (2005).

[47] Singh A Sharmila K Agarwal S. Assessing various strategies used by adolescents to overcome adversity. Asian Pacific J Heal Sci. (2022) 9:221–3. 10.21276/apjhs.2021.9.2.44

[48] Matore MEEM. Pembinaan Instrumen Kecerdasan Menghadapi Cabaran (IKBAR) bagi Pelajar Politeknik Menggunakan Model Rasch. George Town: Universiti Sains Malaysia (2015).

[49] Khairani AZ Abdullah SMS. Relationship between adversity quotient and academic well-being among Malaysian undergraduates. Asian J Sci Res. (2018) 11:51–5. 10.3923/ajsr.2018.51.55

[50] Yazon AD Ang-Manaig K Adrian TWCA. Correlational Study on Mindset, Grit, and Adversity Quotient of Pre-Service Teachers: Evidence in Philippines and Hongkong. Int J Manag Entrep Soc Sci Humanit. (2021) 4:174–81. 10.31098/ijmesh.v4i2.784

[51] Hema G Gupta SM. Adversity Quotient (AQ) for prospective higher education. Int J Indian Psychol. (2015) 2:49–64. 10.25215/0203.080

[52] Grotberg H. A Guide to Promoting Resilience in Children: Strengthening the Human Spirit. Alabama: The Bernard van Leer Foundation (1995).

[53] Sigit DV Suryanda A Suprianti E IchsanIZ. The effect of adversity quotient and gender to learning outcome of high school students. Int J Innov Technol Explor Eng. (2019) 8:34–7. Retrieved from: https://www.ijitee.org/wp-content/uploads/papers/v8i6c2/F10070486C219.pdf

[54] Hairunnisah H Suyitno H Hidayah I. Students mathematical literacy ability judging from the adversity quotient and gender in problem based learning assisted edmodo. Unnes J Math Educ Study. (2019) 8:180–7. Retrieved from: https://journal.unnes.ac.id/sju/index.php/ujmer/article/view/28120

[55] Astri W Latifah L. Pengaruh personal attributes, adversity quotient dengan mediasi self eefficacy terhadap minat berwirausaha. Econ Educ Anal J. (2017) 6:737–51. Retrieved from: https://journal.unnes.ac.id/sju/index.php/eeaj/article/view/20284

[56] Hopkins K. Educational and Psychological Measurement and Evaluation. 8th ed. New York, NY: Pearson (1997).

[57] Kiger LL. Growth Mindset in the Classroom. Empower Res Educ [Internet]. (2017) 1:20–3.

[58] Mansfield CF Beltman S Price A McConney A. “Don't sweat the small stuff:” Understanding teacher resilience at the chalkface. Teach Teach Educ. (2012) 28:357–67. 10.1016/j.tate.2011.11.001

[59] Shen CY. The relative study of gender roles, and job stress and adversity quotient. J Glob Bus Manag Vol. (2014) 10:19–32. Retrieved from: https://kipdf.com/the-relative-study-of-gender-roles-and-job-

[60] Agusta YN. Hubungan Antara Orientasi Masa Depan Dan Daya Juang Terhadap Kesiapan Kerja Pada Mahasiswa Tingkat Akhir Fakultas Ilmu Sosial Dan Ilmu Politik Di Universitas Mulawarman. Psikoborneo. (2014) 2:133–40. 10.30872/psikoborneo.v2i3.3653

[61] Yazon AD Ang-manaig K. Adversity quotient, emotional quotient and academic performance of filipino student- parents PEOPLE. Int J Soc Sci. (2019) 4:1253–64. 10.20319/pijss.2019.43.12531264

[62] Ting SC Tung CY. The job stress-job burnout relationship among junior high school teachers: ambition as a moderator. Int J Educ. (2017) 9:118. 10.5296/ije.v9i4.12321

[63] Harriman L. Measuring Millennials Adversity Quotient ^®^ *and Its Correlation with Individual Performance in Project Teams*. Master Thesis. Coventry: University of Warwick (2016).

[64] Cura JM Gozum JL. A Correlational Study in the Adversity Quotient ^®^ *and the Mathematics Achievement of Sophomore students of College of Engineering and Technology in Pamantasan ng Lungsod ng Maynila*. Bachelor Thesis. Manila: University of the City of Manila (2011).35893275

[65] Halpern DF Benbow CP Geary DC Gur RC Hyde JS Gernsbacher MA. The science of sex differences in science and mathematics. Psychol Sci Public Interes Suppl. (2007) 8:1–51. 10.1111/j.1529-1006.2007.00032.x 25530726 PMC4270278

[66] Lutovac S Kaasila R. Pre-service teachers' future-oriented mathematical identity work. Educ Stud Math. (2014) 85:129–42. 10.1007/s10649-013-9500-8

[67] Kasapoglu K Didin M. Life skills as a predictor of psychological well-being of pre-service pre-school teachers in Turkey. Int J Contemp Educ Res. (2019) 6:70–85. 10.33200/ijcer.544232

[68] Seligman MEP. Helplessness: on depression, development and death. San Francisco: W.H. Freeman (1975).

[69] Stoltz PG. Adversity Quotient: Mengubah Hambatan Menjadi Peluang. 7th ed. Jakarta: PT Grasindo (2007).

