# Supplementary material for: Development of Adversity Quotient (AQ) index of pre-service teachers in Institute of Teacher Education (IPG)
Source: Front Public Health. 2022 Sep 29;10:940323. doi: 10.3389/fpubh.2022.940323 (PMC9557726; doi:10.3389/fpubh.2022.940323)
Supplement: Supplementary file 1 [file Data_Sheet_1.docx]

**LIST OF FIGURES**


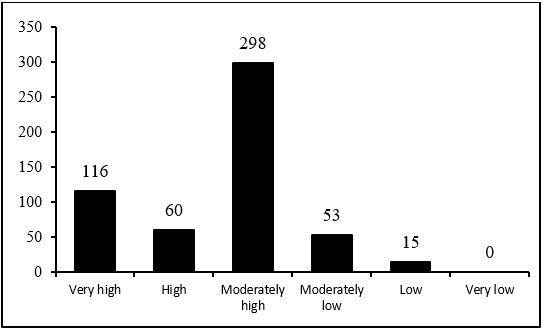


**FIGURE 1** ǀ AQ Levels of Pre-Service Teachers for Control


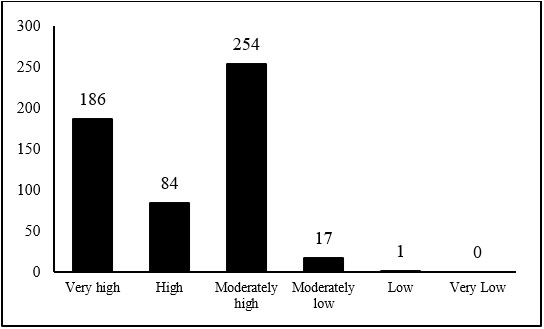


**FIGURE 2** ǀ AQ Levels of Pre-Service Teachers for Ownership


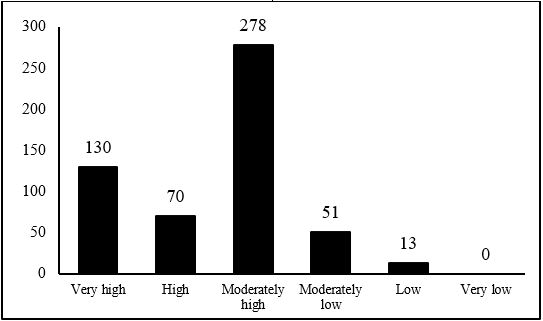


**FIGURE 3** ǀ AQ Levels of Pre-Service Teachers for Reach


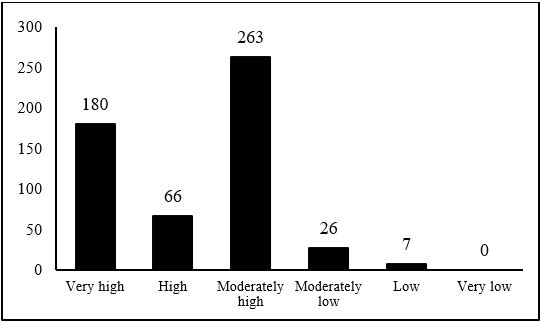


**FIGURE 4** ǀ AQ Levels of Pre-Service Teachers for Endurance


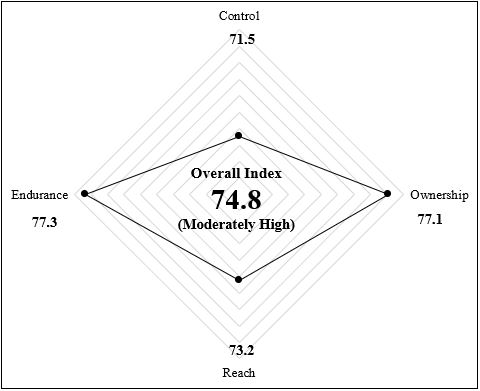


**FIGURE 5 ǀ** Overall AQ Index Score


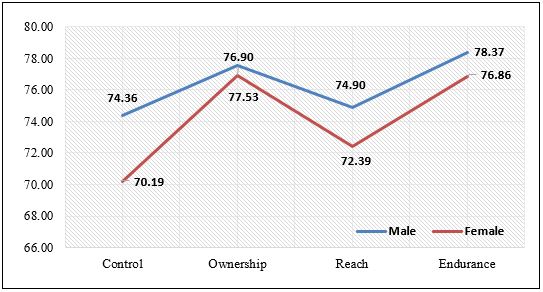


**FIGURE 6 ǀ** AQ Index by Construct Based on Gender

**
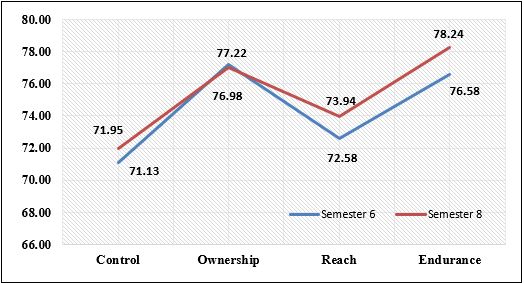
**

**FIGURE 7 ǀ** AQ Index by Construct Based on Semester of Study


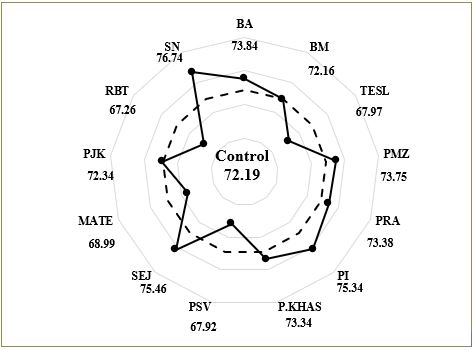


**FIGURE 8 ǀ** AQ Profile Index for Control Construct Based on Field of Study


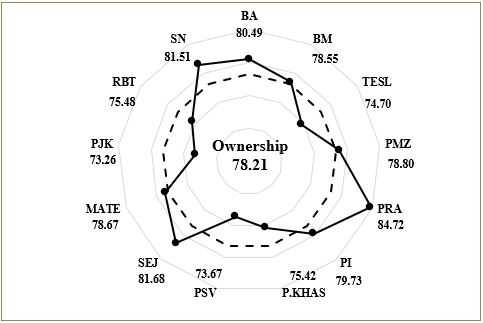


**FIGURE 9 ǀ** AQ Profile Index for Ownership Construct Based on Field of Study


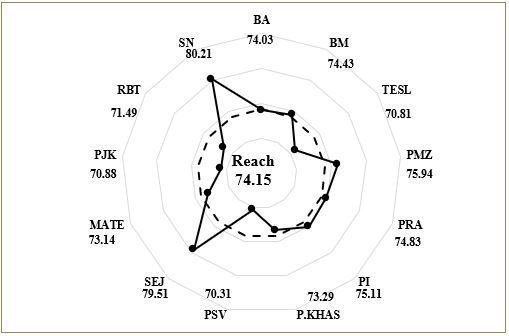


**FIGURE 10 ǀ** AQ Profile Index for Reach Construct Based on Field of Study


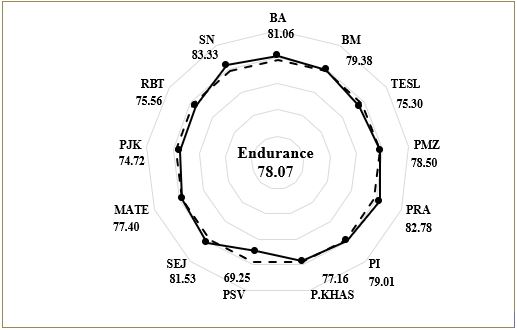


**FIGURE 11 ǀ** AQ Profile Index for Endurance Construct Based on Field of Study
